# The Effect of a Subsequent Dose of Dexmedetomidine or Other Sedatives following an Initial Dose of Dexmedetomidine on Electrolytes, Acid–Base Balance, Creatinine, Glucose, and Cardiac Troponin I in Cats: Part II

**DOI:** 10.3390/vetsci11040143

**Published:** 2024-03-22

**Authors:** Chrysoula Margeti, Georgios Kazakos, Apostolos D. Galatos, Vassilis Skampardonis, Theodora Zacharopoulou, Vassiliki Tsioli, Panagiota Tyrnenopoulou, Epameinondas Loukopoulos, Vasileios G. Papatsiros, Eugenia Flouraki

**Affiliations:** 1 Clinic of Surgery, Faculty of Veterinary Medicine, School of Health Sciences, University of Thessaly, Trikalon 224, 43100 Karditsa, Greece; margeti@uth.gr (C.M.); agalatos@vet.uth.gr (A.D.G.); zacharop@uth.gr (T.Z.); vtsioli@uth.gr (V.T.); ptyrnenop@uth.gr (P.T.); nlouk09@gmail.com (E.L.); 2 Companion Animal Clinic, School of Veterinary Medicine, Aristotle University of Thessaloniki, 54627 Thessaloniki, Greece; gkdvm@vet.auth.gr; 3 Department of Epidemiology, Biostatistics and Animal Health Economics, Faculty of Veterinary Medicine, School of Health Sciences, University of Thessaly, Trikalon 224, 43100 Karditsa, Greece; bskamp@uth.gr; 4 Clinic of Medicine, Faculty of Veterinary Medicine, School of Health Sciences, University of Thessaly, Trikalon 224, 43100 Karditsa, Greece

**Keywords:** acid–base balance, anaesthesia, cardiac troponin I, cat, creatinine, dexmedetomidine, electrolytes, glucose, safety, sedation

## Abstract

**Simple Summary:**

Administration of anaesthetic or analgesic drugs can affect the safety and health of veterinary patients. Dexmedetomidine, a common sedative used in cats, is frequently administered in combination with opioids or other sedative drugs to provide enhanced sedation and reduce the required doses and their side effects. The administration of dexmedetomidine in clinical settings may not always provide the required sedative result; therefore, subsequent administration of an anaesthetic drug may be required. The safety of a drug combination can be assessed using electrolytes, acid–base balance, cardiac troponin I, creatinine, glucose, and other blood parameters. Six adult cats were administered with seven different drug combinations. The first administration for all cats was dexmedetomidine, and the second consisted of either a subsequent dose of dexmedetomidine, an opioid (tramadol, butorphanol, buprenorphine), ketamine, midazolam, or NS 0.9%. The results of our study suggest that the subsequent administration of specific anaesthetic drugs after an inadequate dose of dexmedetomidine did not affect myocardial function, renal function, electrolytes, or acid–base balance. Therefore, a safe level of sedation was achieved. However, the tissue perfusion and oxygenation were reduced, although not below the normal range. Thus, it would be beneficial to support sedated animals with supplemental oxygen. Additionally, the glucose concentration significantly increased, and the haematocrit significantly decreased. Therefore, administration of dexmedetomidine in cats with hyperglycaemia or anaemia is not recommended.

**Abstract:**

The administered dose of dexmedetomidine may occasionally fail to produce the anticipated sedative effects. Therefore, a subsequent dose or administration of another sedative may enhance sedation; however, patient safety may be affected. The safety of seven different drugs administered at the following time point after an insufficient dose of dexmedetomidine was evaluated in a crossover, blind, experimental study that included six healthy adult cats. All cats received an initial dose of dexmedetomidine and a subsequent dose of either dexmedetomidine (Group DD), NS 0.9% (DC), tramadol (DT), butorphanol (DBT), buprenorphine (DBP), ketamine (DK), or midazolam (DM). Animal safety was assessed using repeated blood gas analysis and measurement of electrolytes, glucose, cardiac troponin I, and creatinine to evaluate cardiac, respiratory, and renal function. The median values of creatinine, cardiac troponin I, pH, partial pressure of carbon dioxide, potassium, and sodium did not change significantly throughout the study. Heart rate was significantly decreased in all groups after administration of the drug combinations, except for in the DK group. Respiratory rate decreased significantly after administration of the initial dose of dexmedetomidine and in the DBP and DM groups. The partial pressure of oxygen, although normal, decreased significantly after the administration of dexmedetomidine, whereas the median concentration of glucose increased significantly following the administration of dexmedetomidine. The results of our study suggest that the drug combinations used did not alter the blood parameters above normal limits, while cardiac and renal function were not compromised. Therefore, a safe level of sedation was achieved. However, the administration of dexmedetomidine reduced the partial pressure of oxygen; thus, oxygen supplementation during sedation may be advantageous. Additionally, the increase in glucose concentration indicates that dexmedetomidine should not be used in cats with hyperglycaemia, whereas the decrease in haematocrit suggests that dexmedetomidine is not recommended in anaemic cats.

## 1. Introduction

Alpha_2_-adrenoceptor agonists are frequently used for sedation and analgesia or as preanaesthetic agents in veterinary patients [1,2,3,4,5]. Dexmedetomidine is a highly selective a_2_-agonist that demonstrates dose-dependent and reliable sedation in cats [1,3]. The major concern associated with dexmedetomidine administration is the development of cardiovascular and respiratory depression [2,6]. Similarly, several studies have reported that dexmedetomidine administration may cause metabolic dysregulation and inhibition of the neuroendocrine response [5,7,8,9]. In veterinary practice, dexmedetomidine is often combined with an opioid analgesic or other anaesthetic agent to enhance their collective effects and reduce the required doses [2,10,11].

Administration of commonly used sedative drugs can influence acid–base balance in cats in a dose-dependent manner [5,12]. Several metabolic parameters can be accurately measured in cat venous blood to assess tissue perfusion and the overall acid–base status [13,14]. Maintaining a normal pH is imperative for the optimisation of numerous physiological processes. Therefore, interpretation of the acid–base balance provides useful information regarding the diagnostic procedure and required treatment [14,15]. Similarly, the measurement of the partial pressure of oxygen (pO_2_) and carbon dioxide (pCO_2_) levels in venous blood can help evaluate overall tissue perfusion, as well as metabolic and respiratory conditions [14,16].

The serum and cellular concentrations of sodium (Na^+^) and potassium (K^+^) are closely controlled by the Na^+^-K^+^ pump. While Na^+^ is the main extracellular cation, K^+^ exists primarily intracellularly (95–98%), and serum concentrations are preserved at low levels. Any disturbance in the concentration of these electrolytes can lead to neurological symptoms or conduction disorders such as muscle weakness and cardiac dysrhythmias [17]. Consequently, it is important that plasma Na^+^ and K^+^ concentrations remain within normal levels during the perianaesthetic period [18].

Administration of a_2_-agonists may affect the endocrine system and glucose (Glu) homeostasis [7,19,20]. Administration of dexmedetomidine alone or in combination with other anaesthetic agents can increase blood Glu concentrations and decrease glucagon concentrations [8,21]. Therefore, it is important to determine the clinical implications of these effects. Similarly, when interpreting blood results obtained during sedation, possible alterations in specific blood parameters should be considered [8,21].

Repeated administration of sedative drugs can influence renal function. The administration of a_2_-agonists and other sedative drugs can decrease the renal blood flow and glomerular filtration rate [22,23,24]. Creatinine (CREA) is a renal biomarker often used to determine overall renal function and indirectly measure the glomerular filtration rate (GFR) in veterinary patients [22,25]. Increased plasma CREA concentration is usually an indicator of kidney disease, but the sensitivity of this biomarker is rather low [22,26].

The troponin complex consists of three cardiac troponins which, together, regulate myocardial contraction and are released into circulation after myocardial injury. Cardiac troponin I (cTnI) is responsible for actin–myosin inhibition during calcium shortage; troponin T (cTnT) binds to tropomyosin and binds it, whereas troponin C (cTnC) binds calcium to inhibit contraction [27,28,29]. It has been reported that the administration of a_2_-agonists does not alter [30] or increase the concentration of cTnI [31,32] in small animals. Hence, the effect of dexmedetomidine and other commonly administered sedative drugs on myocardial function can be easily and non-invasively assessed by measuring specific cardiac biomarkers such as cTnI in cats [27,31].

In the clinical setting, the administered dose of dexmedetomidine may occasionally fail to produce the anticipated sedative effect, and an additional dose or an additional drug may be required. The existing literature does not provide sufficient evidence on the impact of subsequent administration of an inadequate dose of dexmedetomidine on the sedation level and safety of drug combinations. Similarly, repeated administration of sedative drugs may influence animal safety. In the present study, after an initial dose of dexmedetomidine, common anaesthetic drugs were administered, and their safety was evaluated using repeated blood gas and electrolyte analyses, as well as cardiac and renal biomarker measurements. The main goal of our study was to determine the effects of routinely used anaesthetic drugs on cardiac, respiratory, and renal function. The main hypothesis of our study was that the use of dexmedetomidine and subsequent administration of additional drugs do not significantly impact the acid–base balance, electrolytes, cardiac troponin I, creatinine, glucose, haematocrit, or haemoglobin concentration, and a safe level of sedation was preserved.

## 2. Materials and Methods

This study is a two-part series of articles. The first part of our article series can be found under the following title: “The effect of dexmedetomidine or other sedatives following an initial dose of dexmedetomidine on sedation and quality of recovery in cats. Part I”. Approval for our study was obtained by the Animal Ethics Committee of the Department of Veterinary Medicine of the University of Thessaly (EDEXZO, number: 138, date: 24 March 2022). In addition, our study was approved by the Animal Ethics Committee of Greece (licence number: 504050, date: 20 December 2021) and complied with the national and EU regulations for animal experimentation (Directive 2010/63/EU).

Six adult domestic shorthaired (DSH) cats, five males and one female, aged 3–4 years and weighing 2.5–4 kg, which were laboratory originated and purpose bred, participated in our study. The animals were housed in the experimentation room throughout the study in calm and quiet cages, distant from other animals. The examiners were familiarised with the animals and practiced their management and handling carefully. All cats were healthy and classified as American Society of Anesthesiologists (ASA) status 1. Before the research commenced, the physical condition of the animals was meticulously evaluated by means of clinical examination, total blood count, and serum biochemical examination. The study did not include any animals that exhibited aggressive or irregular behaviour. The clinical evaluation of the cats, criteria for enrolment, and housing conditions are described in more detail in Part I of the article series.

Each cat participated in the study seven times with seven-day intervals between experiments [6,33]. The study included seven experimental groups that received different drug combinations at specific time points. The first administration consisted of 15 μg/kg dexmedetomidine (Dexdomitor, Orion Pharma, Espoo, Finland). A second administration of one of the additional drugs was performed at the following time point, as presented below. Thus, the second administration was either 10 μg/kg of dexmedetomidine in the DD group; 0,1 mL of saline 0.9% in the DC or control group; 0.2 mg/kg of butorphanol (Dolorex, MSD, Haarlem, The Netherlands) in the DBT group; 20 μg/kg of buprenorphine (Bupaq, Neocell, Athens, Greece) in the DBP group; 2 mg/kg of tramadol (Tramal, Vianex, Athens, Greece) in the DT group; 2 mg/kg of ketamine (Ketaset, Zoetis, Athens, Greece) in the DK group; or 0.1 mg/kg of midazolam (Dormicum, Cheplapharm, Greifswald, Germany) in the DM group. All drugs were administered intramuscularly (IM) to the quadriceps muscle.

In each experiment, four time points were established (T0, T1, T2, and T3), as presented in Table 1. T0 was the time point of the first measurement and administration of dexmedetomidine in all animal groups. Subsequently, the Grint sedation assessment scale was used to establish the following time points (T1 and T2), as described in Part I of the article series. T1 was defined as the time of maximum sedation with dexmedetomidine, and T2 was defined as the point of maximum sedation following the administration of the second drug. At T2, atipamezole was administered to all cats. The time of the conclusion of the experiment was the full recovery time (T3), specifically, the moment when the cat was walking steadily without signs of ataxia.

On the day of the experiment, water and food were withheld for one and ten hours, respectively [34]. At time point T0 and before the administration of any drug, HR and RR were measured by auscultation, and, afterwards, 0.3 mL of venous blood was collected from the jugular vein using a blood gas syringe (Genject Athens, Greece 1 mL-23G). Air bubbles were removed, and the syringe was tightly sealed to achieve anaerobic conditions [16]. Blood was analysed using the I-Stat Alinity handheld blood analyser (CG8+ cartridge). Sample analysis was performed within 2 min of acquisition to prevent parameter alterations [35]. The analyser was used to measure the pH, pO_2_ (mmHg), pCO_2_ (mmHg), Na^+^ (mmol/L), K^+^ (mmol/L), Glu (mg/dL), haematocrit (HCT, %PCV), and haemoglobin (Hb, g/dL). An additional blood sample (0.5 mL) was collected from the jugular vein and transferred into a liquid heparin vial. Within 2 h of collection, the blood was centrifuged at 3000 rpm for 15 min, and serum was collected (approximately 0.2 mL), stored at −20 °C [36], and tested 15–30 days later [37]. Serum was used to measure the cTnI (ng/mL) and CREA (mg/dL) levels. Upon completion of blood sample collection and blood gas analysis, the initial dose of dexmedetomidine was administered. At T1, which was established using the Grint sedation scale, as described in Part I of the series, HR and RR were recorded, and 0.3 mL of venous blood was collected for I-Stat analysis, and the second drug was administered, as determined by animal grouping. At time point T2, HR and RR were recorded, and 0.3 mL of venous blood was collected for blood gas analysis, while 0.5 mL of venous blood was collected for cTnI and CREA evaluation. The a_2_ antagonist atipamezole (Antisedan, Zoetis, Athens, Greece) was administered IM at the dose recommended by the manufacturer [1,38]. For each treatment, three blood samples were collected from each cat.

### Statistical Analysis

G*Power software (version 3.1.9.4) was used to calculate the required sample size following an a priori type of power analysis, conditional on pre-specified levels of significance, power, and effect size. The necessary pre-estimations aimed to investigate the sedative effect, measured on the Grint scale, of the administration of anaesthetic substances, specifically, butorphanol, midazolam, dexmedetomidine, and ketamine, following an insufficient initial sedation with dexmedetomidine. The effect size (Cohen’s *f)* was calculated using the resulting maximum and minimum average differences in Grint scale score between groups, accounting for either minimum, intermediate, or maximum variability in the means’ distribution [39]. The resulting effect sizes (*f* = 0.309, *f* = 0.325, and *f* = 0.437) were used in three power analyses, assuming a repeated measurement design with seven groups, a confidence interval of 95%, a significance level of 5%, and a power of 95%. The required total sample size for each occasion was 35, 28, and 21 animals, achieving powers of 98%, 96%, and 99%, respectively. However, owing to the adopted crossover design of our study, the assignment of the sequence of the seven drug combinations to each of the six cats resulted in 42 observations, satisfying the above maximum requirements of the sample size.

All statistical analyses were performed using Stata 17.0 (Stata Statistical Software, College Station, TX, USA), and the results were interpreted at the 5% level of significance. One of the main concerns of our analyses was the expected dependence of observations from repeated measurements over time within the same animal, and the potential within each different animal–specific drug combination (6 animal × 7 drug combinations = 42 animal–drug combinations), resulting in a three-level hierarchical data structure design.

According to the results of the Shapiro–Wilk tests for normal data and based on the shape of the constructed histograms, the majority of the recorded or measured parameters, although continuous, were not normally distributed.

For most parameters (HR, RR, CREA, cTnI, pO_2_, pCO_2_, GLU, Na^+^, HCT, and Hb), the Wilcoxon signed-rank test was also performed, assessing the change in their median values between time points T0 and T1, T1 and T2, and between T0 and T2 for CREA and cTnI, independently for each drug combination.

For each of the non-normally distributed parameters, a quantile regression model was employed to account for the distributional limitations of parameter divergence from normality, assessing the effect of different drug combinations over T0, T1, and T2. Specifically, the qreg2 command was used to estimate quantile regression, allowing for adjustment of standard errors and t-statistics that are asymptotically valid under heteroskedasticity and intra-cluster correlation between measurements within the same animal and within the same animal treatment combination using the cluster option [40]. The presence or absence of correlation in each potential source of intra-cluster correlation (animal and specific animal–treatment combination level) was tested using the Parente–Santos Silva test [40]. The median values of the non-normally distributed parameters were the dependent variables, while drug combination and administration points (T0, T1, and T2) were the respective independent ones for each corresponding model. The above model structure and specifications were implemented to investigate the effect of the applied drug combinations on all non-normally distributed biochemical and clinical parameters in equally numbered, individual quantile regression models.

K^+^ concentration (W = 0.98993, *p* = 0.5034) and pH (W = 0.98054, *p* = 0.06859) values were normally distributed according to the Shapiro–Wilk test for normal data. Thus, two separate generalised linear mixed-effects regression models were implemented to investigate the effect of the applied treatment combination during the different administration time points since it was presumed that our data were organised in the aforementioned three-level hierarchical structure design. K^+^ concentration and pH value were the dependent variables, whereas drug combination and administration points (T0, T1, and T2) were the independent ones. Additionally, random effect terms at the animal and specific animal–drug combination levels were included to account for the sources of heterogeneity and dependence arising from the multilevel arrangement of observations. The above model structure was implemented to investigate the effects of the applied drug combinations on all normally distributed biochemical and clinical parameters.

## 3. Results

The descriptive statistics of the animals are presented in detail in Part I of the article series.

The normal values of CREA, cTnI, pH, pO_2_, pCO_2_, Glu, Na^+^, Κ^+^, HCT, and Hb in venous blood of cats can be found in Table S1 of the Supplementary Materials. The mean values of pH, Na^+^, and Κ^+^ and the median values of Glu, pO_2_, and pCO_2_ can be found in Table S2 of the Supplementary Materials. The median HR and RR values are presented in Table S3 of the Supplementary Materials.

### 3.1. Heart Rate (HR) and Respiratory rate (RR)

The differences in the median HR values at T2 between groups are presented in Table 2. At T1, the median HR and RR values differed between the groups (Tables S4 and S5 of the Supplementary Materials). The median HR was significantly lower at T1 than at T0 (Coef.: 65, 95% CI: 52.07; 77.93, *p* < 0.001). However, no significant difference was detected in the median HR between T1 and T2 in any group (all *p* > 0.275). The median HR was significantly lower at T2 than at T0 in all groups (all *p* < 0.007), except for the DK group, where the difference was not significant (*p* = 0.083). Comparisons of the median HR values between time points T1 and T2 and T0 and T2 are presented in Table S6 of the Supplementary Materials. Finally, the median HR was significantly lower at T3 than at T0 in the DC (*p* = 0.0313) and DBT groups (*p* = 0.0313). In the DK group, the median HR was higher at T3 than at T0, without a significant difference.

At T2, the median RR was significantly lower in the DBT group than in group DD by 6 bpm (95% CI: 2.93; 9.06, *p* < 0.001), group DT by 9 bpm (95% CI: 2.5; 15.5, *p* = 0.008), and group DM by 9 bpm (95% CI: 1.49; 16.51, *p* = 0.02). Additionally, at T2, the median RR value was significantly lower by 6 bpm in the DBP group than in the DM group (95% CI: 4.08; 7.91, *p* = 0.01).

The median RR was significantly lower by 9 bpm at T1 than at T0 (95% CI: 0.89; 17.11, *p* = 0.03). The median RR was not significantly different between T1 and T2 in all the groups (all *p* > 0.204). In contrast, the median RR showed a statistically significant decrease at T2 compared to T0 in the DBP group of 18 bpm (*p* < 0.001, 95% CI: −25.22; −10.77). The same result was observed between the same time points in the DM group, with a decrease of 15 bpm (*p* = 0.033, 95% CI: −28.62; −1.38). Finally, the median RR was significantly lower at T3 than at T0 in the DC and DM groups (*p* = 0.0313).

**Figure 1:**
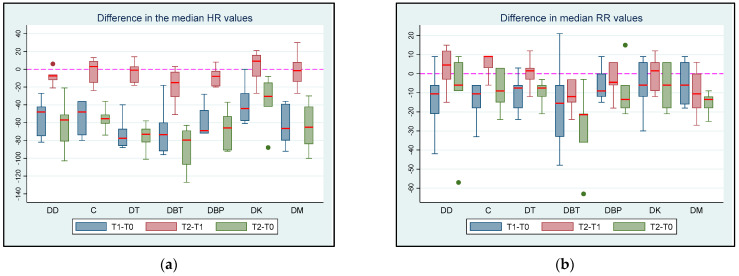
(**a**) HR and (**b**) RR differences between T0 (baseline), T1 (maximum sedation of the first drug), and T2 (maximum sedation of the drug combination) for the seven different groups (DD, DC, DT, D, and DM) of six adult cats that received different drug combinations. Group DD: administration of two subsequent doses of dexmedetomidine; DC: dexmedetomidine-saline 0.9% combination (control group); DT: dexmedetomidine-tramadol combination; DBT: dexmedetomidine-butorphanol combination; DBP: dexmedetomidine-buprenorphine combination; DK: dexmedetomidine-ketamine combination; DM: dexmedetomidine-midazolam combination. The red/horizontal line in the middle of each box plot represents the median value. Dots represent the outliers of the box plots—observations far removed in value from the rest.

The differences of the median HR and RR between the time intervals are presented in Figure 1.

### 3.2. Creatinine and Cardiac Troponin I

At T0, there were no significant differences in the median CREA concentrations between groups (all *p* > 0.068). At T2, the median CREA concentration was significantly higher in the DM group than in the DBT group by 0.37 mg/dL (*p* = 0.046, 95% CI: 0.007; 0.73) and the DK group by 0.49 mg/dL (*p* < 0.001, 95% CI: 0.31; 0.66). There were no significant differences in the median CREA concentrations between T0 and T2 in any of the groups (all *p* > 0.231). CREA concentration remained within normal levels throughout the experiment in all cats.

At T0, there were no significant differences in the median cTnI concentration between the groups (all *p* > 0.093). At T2, the median cTnI concentration was significantly higher by 0.01 ng/mL in the DT group than in the DBP group (*p* = 0.039, 95% CI: 0.019; 0.005). There were no statistically significant differences in the median cTnI concentration at T0 compared with T2 in any of the groups (all *p* > 0.316). The concentration of cTnI remained within normal levels throughout the experiment in all cats.

### 3.3. pH, pO_2_, and pCO_2_

At T0 and T1, the mean pH values did not differ significantly between groups. At T2, the mean pH value was significantly higher in group DM than that in the DT group by 0.062 (*p* = 0.014, 95% CI: 0.012; 0.111) and the DK group by 0.052 (*p* = 0.039, 95% CI: 0.003; 0.102). The mean pH values were significantly higher at T1 than those at T0 by 0.028 (*p* < 0.001, 95% CI: 0.013; 0.044). There was no statistically significant difference in the mean pH values between T1 and T2 in any group (all *p* > 0.101). The mean pH values were significantly higher at T2 than at T0 in the DC (*p* = 0.002), DT (*p* = 0.001), DBP (*p* = 0.018), DK (*p* < 0.001), and DM (*p* = 0.009) groups. More information for the comparison above can be found in the Supplementary Materials (Table S7). All pH values were within normal limits in all groups at all time points.

At T0 and T1, no significant differences in the median pO_2_ values were recorded between the groups. At T2, the median pO_2_ value was significantly lower by 6 mmHg in the DT group than in the DK group (*p* = 0.047, 95% CI: 0.08; 11.91). The median pO_2_ was significantly lower by 5 mmHg at T1 than at T0 (*p* < 0.001, 95% CI: 3.3; 6.7) in the DC group, and the median pO_2_ was significantly lower by 9 mmHg at T1 than at T2 (*p* = 0.018, 95% CI: 1.8; 16.22). In the DT group, the median pO_2_ value was significantly lower by 14 mmHg at T2 than at T0 (*p* = 0.048, 95% CI: 0.15; 27.85). A similar result was observed in the DK group (Coef.: 7, 95% CI: 0.05; 13.95, *p* = 0.049). However, all pO_2_ values remained within the normal limits in all groups at all time points.

At T0, T1, and T2, the median pCO_2_ values were not significantly different between the groups (all *p* > 0.284, *p* > 0.428, and *p* > 0.079, respectively). The median pCO_2_ values were not significantly different between T1 and T0 in any of the groups (all *p* > 0.162). In the DBT group, the median pCO_2_ was significantly higher at T2 than at T1 (*p* < 0.001). The median pCO_2_ value was not significantly different between time points T2 and T1 in the remaining groups (all *p* > 0.263). Accordingly, the median pCO_2_ values were not significantly different between time points T2 and T0 in any group (all *p* > 0.094). Comparisons of the median pCO_2_ values between the time intervals in all groups are shown in Table S8 of the Supplementary Materials.

**Figure 2:**
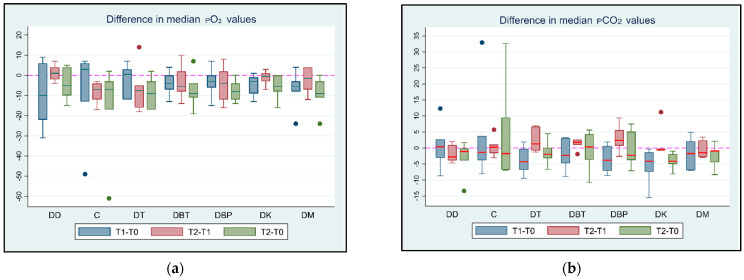
(**a**) pO_2_ and (**b**) pCO_2_ value differences between T0 (baseline), T1 (maximum sedation with the first drug), and T2 (maximum sedation with the drug combination) for seven different groups (DD, DC, DT, DBT, DBP, DK, and DM) of six adult cats that received different drug combinations. Group DD: administration of two repeated doses of dexmedetomidine; DC: dexmedetomidine-NS 0.9% combination (control group); DT: dexmedetomidine-tramadol combination; DBT: dexmedetomidine-butorphanol combination; DBP: dexmedetomidine-buprenorphine combination; DK: dexmedetomidine-ketamine combination; DM: dexmedetomidine-midazolam combination. The red horizontal line in the middle of each box represents the median value. Dots represent the outliers of the box plots—observations far removed in value from the rest.

The differences in median pO_2_ and pCO_2_ values at different time intervals are shown in Figure 2.

### 3.4. Glucose

**Figure 3:**
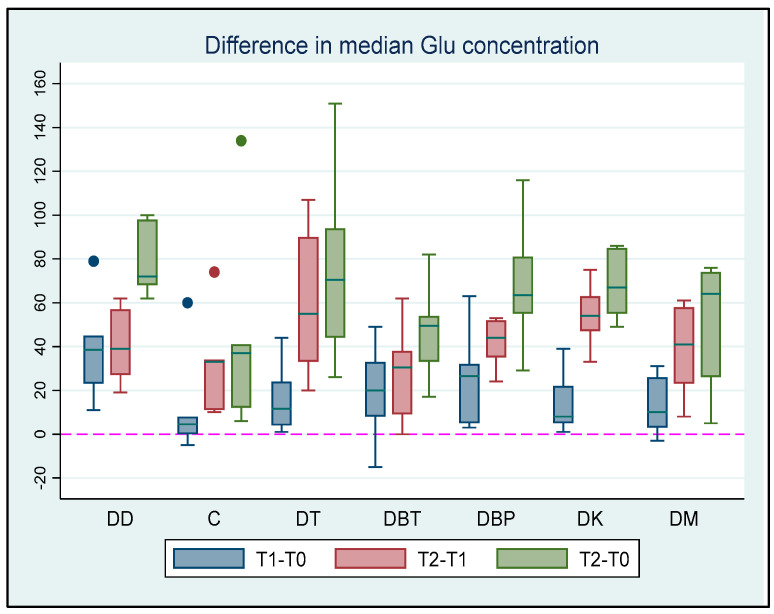
Glu concentration differences among T0 (baseline), T1 (maximum sedation with the first drug), and T2 (maximum sedation with the drug combination) for seven different groups (DD, DC, DT, DBT, DBP, DK, and DM) of six adult cats that received different drug combinations. Group DD: administration of two repeated doses of dexmedetomidine; DC: dexmedetomidine-NS 0.9% combination (control group); DT: dexmedetomidine-tramadol combination; DBT: dexmedetomidine-butorphanol combination; DBP: dexmedetomidine-buprenorphine combination; DK: dexmedetomidine-ketamine combination; DM: dexmedetomidine-midazolam combination. The red horizontal line in the middle of each box represents the median value. Dots represent the outliers of the box plots—observations far removed in value from the rest.

At T0, no significant differences in median Glu concentrations were found between the groups (all *p* > 0.194). At T1, the median Glu concentration was significantly higher in the DD group than in the DK group by 30 mg/dL (*p* = 0.018, 95% CI: 5.47; 54.53) and the DM group by 32 mg/dL (*p* < 0.001, 95% CI: 20.5; 43.5). At T2, the median Glu concentration was significantly higher in the DT group by 45 mg/dL than in the DBT group (*p* = 0.04, 95% CI: 2.07; 87.93). The median Glu concentration showed a statistically significant increase of 23 mg/dL at T1 in comparison to T0 (*p* < 0.001, 95% CI: 13.65; 32.35). The median Glu concentration increased significantly at T2 compared to T1 in all groups (all *p* < 0.045). Finally, the median Glu concentration increased significantly at T2 compared to T0 in all groups (all *p* < 0.002), except in the DC group (*p* = 0.056). More information regarding the comparison above can be found in Table S9 of the Supplementary Materials. The differences in the median Glu values between the time intervals are shown in Figure 3.

### 3.5. Na^+^ and Κ^+^

All K^+^ concentrations were within the normal range at all the time points. The mean K^+^ concentration did not differ significantly between groups at T0 (all *p* > 0.329). At T1, the mean K^+^ concentration was significantly lower in the DD group than in the DC (*p* = 0.016) and DM (*p* = 0.036) groups. The mean K^+^ concentration differences between the groups at T1 can be found in the Supplementary Materials (Table S10). Additionally, the differences in the mean K^+^ concentrations between groups at T2 are presented in Table 3. The mean K^+^ concentration was significantly lower by 0.23 mEq/L at T1 than at T0 (*p* = 0.001, 95% CI: 0.1; 0.36). In the DC group, the mean K^+^ concentration was significantly lower at T1 than at T2 by 0.27 mEq/L (*p* = 0.011, 95% CI: 0.06; 0.47). At T2, the mean K^+^ concentration was significantly lower than at T0 in group DD by 0.53 mEq/L (*p* = 0.001, 95% CI: 0.21; 0.85) and group DK by 0.41 mEq/L (*p* = 0.012, 95% CI: 0.09; 0.73).

**Figure 4:**
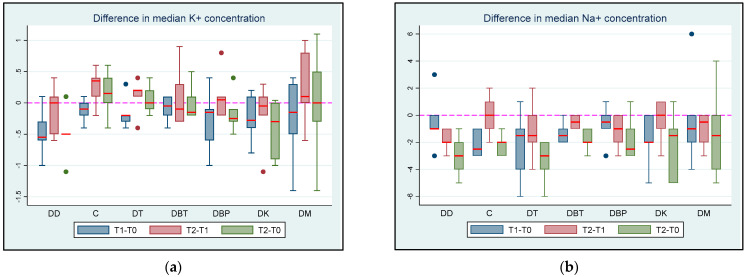
(**a**) K^+^ and (**b**) Na^+^ concentration differences between T0 (baseline), T1 (maximum sedation with the first drug), and T2 (maximum sedation with the drug combination) for seven different groups (DD, DC, DT, DBT, DBP, DK, and DM) of six adult cats that received different drug combinations. Group DD: administration of two repeated doses of dexmedetomidine; DC: dexmedetomidine-NS 0.9% combination (control group); DT: dexmedetomidine-tramadol combination; DBT: dexmedetomidine-butorphanol combination; DBP: dexmedetomidine-buprenorphine combination; DK: dexmedetomidine-ketamine combination; DM: dexmedetomidine-midazolam combination. The red horizontal line in the middle of each box represents the median value. Dots represent the outliers of the box plots—observations far removed in value from the rest.

All Na^+^ concentrations were within the normal range at all time points except at T2, when a cat in the DT group had a Na^+^ concentration of 142 mEq/L. At T0, the median Na^+^ concentration was significantly lower in the DM group than in the DK group by 3 mEq/L (*p* = 0.045, 95% CI: 0.074; 5.92). There were no significant differences in the median Na^+^ concentrations between the groups at T1 (all *p* > 0.092) and T2 (all *p* > 0.132). A significant reduction (*p* < 0.001) was observed in the median Na^+^ concentration when T0 was compared to T1. Accordingly, a significant reduction (*p* = 0.0313) in the median Na^+^ concentration was observed in the DC, DT, and DBT groups when T0 was compared to T2. The differences in median K^+^ and Na^+^ concentrations at different time intervals are shown in Figure 4.

### 3.6. Haematocrit and Haemoglobin

At T0, the HCT and Hb values were within the normal range for all the cats. There were no significant differences in the median HCT values between groups at T0 (all *p* > 0.642), T1 (all *p* > 0.297), or T2 (all *p* > 0.274). There were no statistically significant differences in the median HCT between T1 and T0 in all groups (all *p* > 0.0625), except for the DBT and DK groups, where the mean HCT was significantly lower (*p* = 0.0313). No statistically significant difference in median HCT concentration was observed between T1 and T2 (all *p* > 0.0938). The median HCT was significantly lower at T2 than at T0 in the DC, DT, DBT, DBP, and DK groups (all *p* = 0.0313), whereas no statistically significant difference between T2 and T0 was observed in the DD and DM groups (*p* = 0.0625).

There were no significant differences in the median Hb concentrations between groups at T0 (all *p* > 0.671), T1 (all *p* > 0.603), and T2 (all *p* > 0.092). The median Hb concentration was significantly lower at T1 than at T0 in the DBT, DBP, and DK groups (*p* = 0.0313); however, no statistically significant differences were observed in the rest of the groups (all *p* > 0.0625). For all groups, there was also no statistically significant difference in the median Hb value between T1 and T2 (all *p* > 0.0625). The median Hb concentration was significantly lower at T2 than at T0 in the DC, DT, DBT, DBP, and DK groups (*p* = 0.0313); however, no statistically significant difference was observed for the DD and DM groups (*p* = 0.0625).

**Figure 5:**
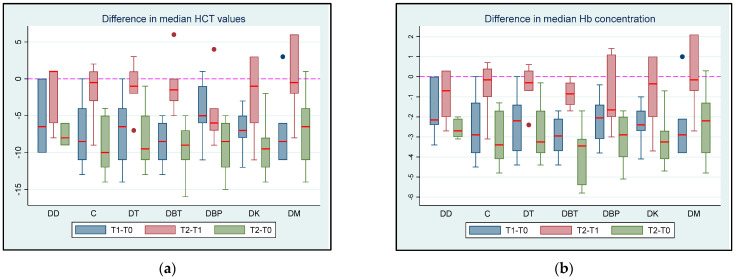
(**a**) HCT values and (**b**) Hb concentration differences between T0 (baseline), T1 (maximum sedation of the first drug), and T2 (maximum sedation of the drug combination) for seven different groups (DD, DC, DT, DBT, DBP, DK, and DM) of six adult cats that received different drug combinations. Group DD: administration of two repeated doses of dexmedetomidine; DC: dexmedetomidine-NS 0.9% combination (control group); DT: dexmedetomidine-tramadol combination; DBT: dexmedetomidine-butorphanol combination; DBP: dexmedetomidine-buprenorphine combination; DK: dexmedetomidine-ketamine combination; DM: dexmedetomidine-midazolam combination. The red horizontal line in the middle of each box represents the median value. Dots represent the outliers of the box plots—observations far removed in value from the rest.

The differences in the median HCT concentrations and Hb values between time intervals are shown in Figure 5a (HCT values) and Figure 5b (Hb concentration differences) between T0 (baseline), T1 (maximum sedation of the first drug), and T2 (maximum sedation of the drug combination) for seven different groups (DD, DC, DT, DBT, DBP, DK, and DM) of six adult cats that received different drug combinations.

The comparison of the median HCT values and Hb concentrations for all groups between the time intervals can be found in the Supplementary Materials (Tables S11 and S12).

## 4. Discussion

### 4.1. Creatinine, Cardiac Troponin I, and Heart Rate (HR)

In our study, CREA concentrations were within the normal limits in all groups. It is important to emphasise that no drug combination had a statistically significant effect on the CREA concentration. However, at T2, the median CREA concentration was significantly higher in the DM group than that in the DBT and DK groups. These differences, although statistically significant, are of little clinical importance considering that CREA concentrations remained within normal levels. Similarly, it has been documented that CREA values do not significantly change after the administration of ketamine–dexmedetomidine, ketamine–dexmedetomidine-butorphanol [41], or ketamine–midazolam [26,41] combinations in cats. Plasma CREA concentration is a commonly used biomarker for evaluating renal function in cats [42]. The CREA concentrations in the present study remained within the normal range; therefore, it appears that renal function was not compromised by the administration of the drug combinations. However, it has been reported that CREA may not increase above the normal range in mild renal dysfunction; thus, it has limited sensitivity [25]. Consecutively, the concurrent measurement of symmetric dimethylarginine (SDMA) may be a more sensitive indicator of renal dysfunction [43].

Troponins are released by the myocardium in proportion to tissue injury and myocardial dysfunction [27,29]. Measurement of cTnI levels in the blood plasma helps monitor cardiac function in critically ill patients [28]. In our study, cTnI concentrations were not significantly altered after administration of the drug combinations, and all mean values remained within the normal range. However, at T2, the median cTnI value was significantly higher in the DT group than in the DBP group, which was not clinically important considering that all values remained within the normal range. Regarding cTnI values, normal myocardial function was not affected, and no significant myocardial injury was induced by the combination of drugs administered during the assessment period of the present study.

A_2_-agonists increase systemic vascular resistance (SVR) and decrease sympathetic tone, resulting in a decrease in HR [34,44]. Additionally, cardiovascular depression due to opioids develops through vagal tone increases [21]. In our study, a significant decrease in HR was observed in all groups after administration of dexmedetomidine, as expected. Furthermore, at T2 in the DC group, the HR was significantly higher than that with the combination of dexmedetomidine with opioids in the DT, DBT, and DBP groups. This was most likely the result of the synergistic effects of dexmedetomidine and opioids. In contrast, in the DK group, the median HR value was not significantly different at T2 compared to that at T0. Likewise, it has been reported that ketamine administration increases HR, cardiac output, oxygen demand, and arterial blood pressure [5]; therefore, it may partially balance the bradycardic effects of dexmedetomidine [2]. In our study, 15 min after atipamezole administration (T2), the HR in all the cats was >100 bpm. These results are in accordance with the literature, where it has been reported that the administration of atipamezole reverses the effects of a_2_-agonists within five minutes [1]. However, HR may remain low for up to five hours if no atipamezole is administered [6]. The potential for mild, transient bradycardia resulting from the use of a_2_-agonists alone or in combination with opioids should always be considered by clinicians [3].

In our study, although mild bradycardia was observed, the HR remained ≥ 88 bpm at T1 and ≥81 bpm at T2 in all cats, and no intervention was deemed necessary. Likewise, cTnI concentrations were not significantly altered after administration of the drug combinations; therefore, bradycardia did not produce any apparent myocardial injury. Transient mild bradycardia has been reported after dexmedetomidine administration in dogs, and no intervention was required [45]. Similarly, in a crossover study in healthy cats by Diggelmann et al. (2023), the administration of a high dose of medetomidine (100 μg/kg) in cats resulted in marked bradycardia and reduction in cardiac output but not a significant increase in cTnI concentrations [30]. In contrast, the administration of 40 μg/kg dexmedetomidine IM in healthy cats by Côté et al. (2022) resulted in similar effects as in the study by Diggelmann et al.; however, serum cTnI concentrations were significantly increased [31]. According to previous reports, the HR level returns to normal values 10–15 min after the administration of atipamezole [1,46]. Nonetheless, if no atipamezole is administered, the effects of dexmedetomidine on HR may persist for 3–5 h [1,6]. In our study, it was observed that, at the time of full recovery, the HR had not returned to normal values in any group, except for group DK. In this group, the median HR was higher at T3 than at T0, although the difference was not significant. It should be noted that these results may have been statistically different if a larger sample size was used.

### 4.2. pH, pO_2_, pCO_2_, and Respiratory Rate (RR)

Notably, the pH in our study did not exceed the normal range (>7.46) [47] after administration of the drug combinations. The increase in pH following administration of the drug combinations in the present study was not deemed clinically important. The results of our study are in contrast to the study by Pypendop (2011), where the arterial pH significantly decreased after the administration of dexmedetomidine in isoflurane-anaesthetised cats [12]. In that study, the result was attributed to an increase in pCO_2_ and decrease in arterial bicarbonate concentration. Likewise, in a study in humans, intravenous infusion of dexmedetomidine induced a statistically significant, dose-dependent decrease in pH but without any clinical importance [48]. Nevertheless, the effect of the administration of specific anaesthetic drugs on pH levels requires further investigation.

In cats, venous blood gases are used to assess ventilation but not oxygenation [49]. In the present study, a significant decrease in the median pO_2_ was observed at T1 after dexmedetomidine administration. Similarly, in a study by Pypendop (2011), when dexmedetomidine was administered to isoflurane-anaesthetised cats, a decrease in mixed venous pO_2_ was recorded, while the arterial pO_2_ and the O_2_ extraction ratio increased [12]. This result was indicative of a possible imbalance between O_2_ delivery and consumption, attributed to reduced cardiac output [12]. The decrease in the median pO_2_ value in our study presumably occurred because of the depressant effect of a_2_-agonists on the circulatory and respiratory systems, as has been reported in cats [1,2,5,12] and dogs [34,44]. Therefore, O_2_ supplementation may be beneficial when dexmedetomidine is administered to avoid tissue hypoxia.

In the DT and DK groups, the median pO_2_ values significantly decreased at T2 compared with T0, although a concurrent significant decrease in RR was not observed. This result is probably attributable to respiratory depression caused by these drugs [5,50]. As tramadol is a low-affinity μ-agonist, the respiratory depression is considered minimal [50]. In a study by Teppema et al. (2003), after IV administration of tramadol (1–4 mg/kg) in cats, a dose-dependent depression of ventilatory control was observed owing to the μ-receptor agonistic effect of the drug [50]. This depression of ventilatory control was expressed as a decrease in CO_2_ sensitivity and an increase in the apnoeic threshold. Moreover, the use of ketamine appears to increase cardiac workload and oxygen demand [5]. In our study, administration of tramadol or ketamine after an initial dose of dexmedetomidine resulted in decreased venous pO_2_ values. Therefore, supplementary O_2_ administration is recommended when a combination of these drugs is administered, even when a significant decrease in the RR is not observed. In contrast, in the DC group, the median pO_2_ was significantly higher at T2 than at T1. Thus, the decrease in the pO_2_ value by the administration of a single dose of dexmedetomidine was transient, and, by time point T2, an increase in the pO_2_ level was already instigated.

The pCO_2_ value remained within normal levels in the present study at all time points (25–42 mmHg) [47], except for in two cats in the DBP group at T2, when pCO_2_ was >42 mmHg. Nevertheless, the alteration in venous pCO_2_ levels in the current study was not clinically important. In a study by Pypendop (2011), when dexmedetomidine was administered to isoflurane-anaesthetised cats, mixed venous and arterial pCO_2_ values significantly increased [12]. Similarly, a dose-dependent suppression in the response to CO_2_, and therefore a decreased respiratory response to hypercapnia, was detected after intravenous administration of 10 μg/kg dexmedetomidine in dogs [51]. The mild increase in pCO_2_ observed in our study may be an indication of respiratory compensation for the detected increase in pH. However, it should be noted that the compensatory responses in cats have not been fully elucidated as they have not been extensively studied as compared to dogs. Therefore, it is generally assumed that the responses of these two species are similar [15].

In the present study, the RR values significantly decreased after the administration of dexmedetomidine compared to baseline measurements (T0). This RR reduction was associated with a concurrent decrease in pO_2_ values after dexmedetomidine administration; thus, considerable depression in ventilation was present. Similarly, it has been reported that a_2_-agonists cause respiratory depression through a_2_-adrenoceptor stimulation and CNS depression [44]. Previous research has yielded conflicting results regarding the effect of dexmedetomidine on RR. While some studies have failed to observe any significant decrease in RR [2], others have reported a temporary, minor reduction in RR following the administration of dexmedetomidine [1,2,6].

The most important adverse effect of opioids is respiratory depression caused by μ-opioid receptor agonism [52]. In the present study, only a partial μ-agonist, buprenorphine, was used. Although it is not a full μ-agonist, a significant reduction in RR was recorded in the DBP group, probably owing to the synergistic effect of dexmedetomidine and buprenorphine. Similarly, it has been reported that the combination of dexmedetomidine and buprenorphine significantly reduces RR in cats [53,54]. In contrast, buprenorphine alone (10 μg/kg) did not significantly decrease RR in the same species [55]. In the present study, a significant reduction in RR was observed in the DM group. This is in accordance with the literature, where it has been reported that midazolam can decrease RR in cats [26,56], probably through CNS depression and muscle relaxation [57]. Furthermore, in a study by Castro et al. (2022), the administration of a dexmedetomidine–midazolam–methadone combination in healthy cats resulted in marked respiratory depression, described as a reduction in RR and arterial pO_2_ and an increase in arterial pCO_2_ [58].

### 4.3. Glucose

A transient increase in blood Glu or stress hyperglycaemia can occur in sick or stressed cats [59]. Concurrently, a_2_-agonists cause metabolic dysregulation via direct insulin suppression [7]. Dexmedetomidine inhibits insulin secretion in rats predominantly through peripheral (pancreatic) a_2_-adrenoceptors and secondarily through the activation of imidazoline receptors [60]. It has been reported that 10 μg/kg dexmedetomidine significantly increases Glu concentration and decreases insulin concentration in cats [8,61]. In accordance with the above results, in our study, the median Glu concentration increased significantly after the administration of dexmedetomidine.

The median Glu concentration increased significantly in the DK group between T1 and T2 and between T0 and T2. Similarly, plasma Glu concentrations have been shown to increase after administration of a combination of dexmedetomidine and ketamine in cats [41]. A possible explanation for this result is that anaesthetic drugs such as ketamine or propofol affect the subcortical pathway, causing an increase in glucocorticoid secretion, thus increasing the relocation of Glu from tissues to circulation [62].

In the present study, Glu concentration was also significantly increased in the DM group between T0 and T2 and between T1 and T2. In contrast to our findings, previous research has suggested that the use of midazolam alone (at a dose of 0.5 mg/kg) or in combination with medetomidine did not result in a substantial increase in Glu concentration. However, medetomidine administration alone caused a notable increase in the Glu levels [63]. These results suggest that midazolam can reduce the hyperglycaemic effect of a_2_-agonists. The mechanism of this action is unknown, but it is possibly a result of interactions between a_2_-adrenoceptors and benzodiazepine receptors, or between γ-aminobutyric acid (GABA) and hyperglycaemic mechanisms [63]. In our study, midazolam was administered at a low dose (0.1 mg/kg). Therefore, it is possible that the administration of a larger dose might have different effects on plasma Glu concentrations.

Additionally, in our study, the median Glu concentration increased in the DT group between T0 and T2 and between T1 and T2. The reported effects of tramadol on plasma Glu concentration vary. Tramadol has been reported to activate opioid receptors [64], generate dose-dependent hypoglycaemic effects [65,66,67], activate serotonin receptors, and increase muscle Glu uptake [68]. In contrast, several studies have reported hyperglycaemia following tramadol administration in cats [69], dogs [70], and rabbits [71]. The hyperglycaemic effects of tramadol may arise through activation of a_2_-adrenergic receptors [72]. In our study, in the DT group, it was not clear whether the Glu concentration increased only through the action of dexmedetomidine or through a probable synergistic hyperglycaemic action of dexmedetomidine and tramadol. However, it is possible that the administered dose of tramadol was insufficient to mitigate the hyperglycaemic effects of dexmedetomidine.

### 4.4. Na^+^ and K^+^

Plasma Na^+^ and K^+^ concentrations are closely controlled by homeostatic mechanisms to prevent life-threatening alterations [18,73]. In the present study, we detected a significant reduction in the mean K^+^ concentration in all groups after dexmedetomidine administration (T0–T1) and in the median Na^+^ concentration in the DC, DT, and DBT groups after the administration of the drug combinations (T0–T2), whereas the median Glu concentration significantly increased in all groups after the administration of dexmedetomidine. These findings are in contrast with those reported in the literature. Numerous studies have reported that K^+^, Na^+^, and Glu concentrations are codependent, and that a decrease in insulin concentration leads to impairment of the Na^+^-K^+^ pump, which causes a gradual influx of Na^+^ into and efflux of K^+^ out of the cells [73,74]. Additionally, when K^+^ initiates cell depolarisation, insulin secretion commences from pancreatic β cells, and stimulation of insulin receptors leads to increased activity of the Na^+^-K^+^ pump [75]. In the current study, the increase in Glu concentration was not accompanied by an increase in K^+^ concentration. In contrast, the median pH increased in most groups, whereas the mean K^+^ concentration decreased. In accordance with the above results, it has been reported that metabolic alkalosis initiates the movement of K^+^ into the cell, whereas metabolic acidosis initiates the movement of K^+^ out of the cell [76]. This result is also in accordance with a study by Ha et al. (2013), in which hypokalaemia occurred more frequently during metabolic alkalosis in dogs and cats [77]. Similarly, stress generated during the perianaesthetic period increases catecholamine levels [78], which influences the rapid distribution of K^+^ into the intracellular space [76]. It has also been reported that, when hypokalaemia occurs, H^+^ ions are transported into the renal tubular cells in exchange for K^+^ ions, resulting in deterioration of pre-existing metabolic alkalosis [79]. In the present study, although neither alkalosis nor hypokalaemia was present, our results may demonstrate an inclination toward a decrease in K^+^ concentration as the pH increased.

### 4.5. Haematocrit and Haemoglobin

In the present study, the median HCT and Hb values decreased after administration of the drug combinations in all groups, except in groups DD and DM, where a non-significant reduction was observed. A possible explanation for the reduction in HCT and Hb values after the administration of sedative drugs has been documented by Adetunjia et al. (2000) [80]. This study reported a reduction in splenic response and a consecutive reduction in Hb concentration after the administration of sedative drugs or after a reduction in sympathetic activity [80]. These changes probably occur during the perianaesthetic period due to sympathetic system suppression, decreased systemic vascular resistance (SVR), vasodilation, and haemodynamic imbalance. These alterations result in increased concentrations of red blood cells in the spleen and several vascular reservoirs [41,81]. Similar to our study, HCT has been reported to significantly decrease after dexmedetomidine administration [82]. Additionally, Hb concentration decreased after a dexmedetomidine–methadone combination with or without midazolam in cats [21,58].

The estimated feline blood volume ranges from 37 to 67 mL/kg [83,84]. In the present study, 1.9 mL of venous blood was collected for each experiment. In the smallest cat included in the study (2.5 kg body weight), with a mean value of 60 mL/kg, the total blood volume was approximately 150 mL. Thus, for each experiment, approximately 1.26% of the total blood volume or 0.76 mL/kg of blood was collected, suggesting a total blood collection of <5.5 mL/kg throughout the entire study. Furthermore, at T0, all cats had normal HCT and Hb concentrations before the start of each experiment. In a study by Zeiler et al. (2020), healthy cats were anaesthetised with buprenorphine, alfaxalone, and isoflurane, and controlled blood loss was generated (4.5 ± 1.1 mL/kg of blood), resulting in a mean HCT reduction of 0.00 (±0.01) L/L or −1.8 (±3.1)% and a mean Hb reduction of 3 (±2) mg/dL or −3.2 (±2.5)% [85]. The amount of blood collected from the cats in our study did not interfere with HCT and Hb concentrations.

### 4.6. Limitations

This study has several limitations. The sample size was determined through a power analysis based on changes in sedation scale scores which were analysed in Part I of the article series. As a result, the power for assessing the differences in other parameters remains undetermined. However, the small sample size may have led to an underestimation of significant differences between the treatment groups.

Stress levels in cats can lead to changes in several parameters, such as HR, RR, HCT, or Glu (stress hyperglycaemia). In the current study, these effects were attenuated because the animals were acclimated to the environment and the researchers. The amount of stress developed was similar in each experiment. Nonetheless, similar modifications may occur in the clinical environment. Consequently, our findings may be applicable in a clinical setting.

## 5. Conclusions

While the median pO_2_ remained within normal limits, a statistically significant decrease was observed following consecutive doses of dexmedetomidine and other anaesthetic drugs. Therefore, O_2_ supplementation may be beneficial when these drug combinations are administered to prevent tissue hypoxia. Furthermore, a single dose of dexmedetomidine in the control group produced a transient decrease in pO_2_ which was resolved by the end of the experiment. In contrast, the venous pH and pCO_2_ remained within normal ranges throughout the experiment and were not affected by the administered drug combinations. The drug combinations in our study did not alter the cTnI level of the animals and thus did not significantly affect the myocardial function of the animals. CREA concentration remained within normal levels throughout the study; therefore, the drug combinations used did not interfere with renal function. The administration of dexmedetomidine resulted in a significant increase in the median concentration of Glu in all the study groups, and this increase was not mitigated by using additional medications. Consequently, the administration of dexmedetomidine or a combination of dexmedetomidine and tramadol is not recommended in cats with pre-existing hyperglycaemia as it could worsen this result. In contrast, the median K^+^ and Na^+^ concentrations remained within normal levels, although the median K^+^ concentration significantly decreased after drug administration. Additionally, after drug combination treatment in our study, we observed a decrease in the median HCT and Hb values in most groups. Therefore, the use of these drug combinations is probably not indicated in cats with anaemia in clinical settings.

## Figures and Tables

**Table 1:** Timeline of the study.

Time Point	Measurements and Administrations
T0	Measurement of HR and RR Venous blood collection (blood gas, electrolytes, Glu, CREA, cTnI) Administration of dexmedetomidine
T1	Measurement of HR and RR Venous blood collection (blood gas, electrolytes) Administration of second drug
T2	Measurement of HR and RR Venous blood collection (blood gas, electrolytes, Glu, CREA, cTnI) Administration of atipamezole
T3	Measurement of HR and RR Full recovery

Glu: glucose, CREA: creatinine, cTnI: cardiac troponin I.

**Table 2:** Comparison of the median HR values at time point T2 (maximum sedation of the drug combination) between seven groups of adult cats that received different drug combinations.

Group	DD (*p* Value)	DC (*p* Value)	DT (*p* Value)	DBT (*p* Value)	DBP (*p* Value)	DK (*p* Value)
DC	Coef.: 36 (0.001)					
DT	Coef.: 15 (0.02)	Coef.: −21 (0.001)				
DBT	(0.497)	Coef.: −33 (<0.001)	Coef.: 12 (0.009)			
DBP	Coef.: 21 (0.004)	Coef.: 15 (0.019)	Coef.: 6 (<0.001)	Coef.: 18 (0.001)		
DK	Coef.: 36 (0.011)	(1.00)	(0.128)	Coef.: 33 (0.032)	(0.298)	
DM	(0.103)	Coef.: 18 (0.04)	(0.722)	(0.128)	(0.743)	(0.158)

If the difference was statistically significant, then the co-efficiency (Coef.) value is also presented. A Coef. > 0 indicates that the HR value was significantly higher for the group in the row than that for the group in the column. A Coef. < 0 indicates that the HR value was significantly lower for the group in the row than for the group in the column. All confidence intervals were set at 95%. The *p* values are presented in the parentheses. Group DD: administration of two repeated doses of dexmedetomidine; DC: dexmedetomidine-NS 0.9% combination (control group); DT: dexmedetomidine-tramadol combination; DBT: dexmedetomidine-butorphanol combination; DBP: dexmedetomidine-buprenorphine combination; DK: dexmedetomidine-ketamine combination; DM: dexmedetomidine-midazolam combination.

**Table 3:** Comparison of the mean K^+^ concentrations at time point T2 between seven groups of adult cats that received different drug combinations.

Group	DD (*p* Value)	DC (*p* Value)	DT (*p* Value)	DBT (*p* Value)	DBP (*p* Value)	DK (*p* Value)
DC	Coef.: 0.77 (0.001)					
DT	(0.05)	(0.119)				
DBT	Coef.: 0.035 (0.035)	(0.161)	(0.876)			
DBP	Coef.: 0.49 (0.029)	(0.185)	(0.815)	(0.938)		
DK	(0.794)	Coef.: −0.71 (0.001)	(0.073)	(0.051)	Coef.: −0.43 (0.043)	
DM	Coef.: 0.67 (0.003)	(0.640)	(0.275)	(0.350)	(0.391)	Coef.: 0.62 (0.004)

If the difference is statistically significant, then the co-efficiency (Coef.) value is also presented. A Coef. > 0 indicates that the K^+^ concentration was significantly higher for the group in the row than that for the group in the column. A Coef. < 0 indicates that the K^+^ concentration was significantly lower for the group in the row than for the group in the column. All confidence intervals were set at 95%. The *p* values are presented in the parentheses. K^+^ concentration is measured in mEq/L. T2: maximum sedation with the drug combination and administration of atipamezole. Group DD: administration of two repeated doses of dexmedetomidine; DC: dexmedetomidine-NS 0.9% combination (control group); DT: dexmedetomidine-tramadol combination; DBT: dexmedetomidine-butorphanol combination; DBP: dexmedetomidine-buprenorphine combination; DK: dexmedetomidine-ketamine combination; DM: dexmedetomidine-midazolam combination.

## Data Availability

The data is available upon request from the corresponding author.

## Supplementary Materials

The following supporting information can be downloaded at: https://www.mdpi.com/article/10.3390/vetsci11040143/s1, Table S1. Normal concentrations of CREA, cTnI, pH, pO_2_, pCO_2_, Glu, Na^+^, Κ^+^, HCT, and Hb in venous blood of cats. Table S2. Mean (±SD) pH values, K^+^ and Na^+^ concentrations, median (range) Glu concentrations, and median (range) pO_2_ and pCO_2_ values at T0, T1, and T2 for the seven groups of six adult cats that received different drug combinations. Table S3. Median (range) HR and RR at T0, T1, T2, and T3 for the seven groups of six adult cats that received different drug combinations. Table S4. Comparison of the median HR values at time point T1 between the seven groups of adult cats that received different drug combinations. Table S5. Comparison of the median RR values at time point T1 between the seven groups of adult cats that received different drug combinations. Table S6. Comparison of the median HR values between time points T1 and T0 and between T0 and T2 in the seven groups of adult cats that received different drug combinations. Table S7. Comparison of the median pH values between T0 and T2 in seven groups of adult cats that received different drug combinations. Table S8. Comparison of the median pCO_2_ values between the time intervals in the seven groups of adult cats that received different drug combinations. Table S9. Comparison of the median Glu values between T1 and T2 and between T0 and T2 in the seven groups of adult cats that received different drug combinations. Table S10. Comparison of the mean K^+^ concentrations at T1 between the seven groups of adult cats that received different drug combinations. Table S11. Comparison of the median HCT values between the time intervals in the seven groups of adult cats that received different drug combinations. Table S12. Comparison of the median Hb concentrations between the time intervals in the seven groups of adult cats that received different drug combinations.
