# Supplementary material for: The Effect of a Subsequent Dose of Dexmedetomidine or Other Sedatives following an Initial Dose of Dexmedetomidine on Electrolytes, Acid–Base Balance, Creatinine, Glucose, and Cardiac Troponin I in Cats: Part II
Source: Vet Sci. 2024 Mar 22;11(4):143. doi: 10.3390/vetsci11040143 (PMC11053990; doi:10.3390/vetsci11040143)
Supplement: Supplementary file 1 [file vetsci-11-00143-s001.zip › vetsci-2877045-supplementary.pdf]

*veterinary sciences*

MDPI

*Supplementary Materials*

# The Effect of a Subsequent Dose of Dexmedetomidine or Other Sedatives following an Initial Dose of Dexmedetomidine on Electrolytes, Acid–Base Balance, Creatinine, Glucose, and Cardiac Troponin I in Cats: Part II

**Chrysoula Margeti, Georgios Kazakos, Apostolos D. Galatos, Vassilis Skampardonis, Theodora Zacharopoulou, Vassiliki Tsioli, Panagiota Tyrnenopoulou, Epameinondas Loukopoulos, Vasileios G. Papatsiros and Eugenia Flouraki**

**Table S1.** Mean (± Standard Deviation - SD) pH values, median (range) Glu concentrations, $pO_2$ and $pCO_2$ at T0, T1, and T2 for the seven groups of six adult cats that received different drug combinations.

| | | pH | | | Glu | |
|---|---|---|---|---|---|---|
| **Groups** | **T0** | **T1** | **T2** | **T0** | **T1** | **T2** |
| DD | 7.36 (±0.1) | 32.8 (±5.7) | 34 (±3.1) | 86.5 (81-90) | 103.5 (74-149) | 162 (145-188) |
| DC | 7.31 (±0.05) | 39.2 (±3.2) | 30.1 (±6.6) | 92.5 (79-107) | 127.5 (96-160) | 128 (85-223) |
| DT | 7.27 (±0.09) | 37 (±7.8) | 30.7 (±7.4) | 86.5 (83-97) | 103 (89-128) | 160 (109-235) |
| DBT | 7.31 (±0.09) | 36.8 (±7.6) | 33.5 (±8.5) | 87.5 (58-100) | 103 (85-122) | 136 (105-147) |
| DBP | 7.33 (±0.08) | 34.7 (±10.7) | 30.3 (±2.7) | 91 (82-94) | 116.5 (85-153) | 156 (123-206) |
| DK | 7.27 (±0.03) | 35.8 (±3.9) | 34.7 (±2.9) | 89 (83-90) | 94.5 (91-126) | 156 (139-173) |
| DM | 7.35 (±0.04) | 36.3 (±4.7) | 34.2 (±4.6) | 87 (80-91) | 93.5 (88-116) | 147.5 (96-166) |
| | | **$pO_2$** | | | **$pCO_2$** | |
| **Groups** | **T0** | **T1** | **T2** | **T0** | **T1** | **T2** |
| DD | 43 (29-56) | 34.5 (22-38) | 34 (29-37) | 34.4 (25-48.2) | 36.4 (29.4-39.5) | 33.5 (30.3-34.8) |
| DC | 39 (32-70) | 39 (35-44) | 32.5 (21-38) | 40.4 (30.7-46.5) | 35.7 (32.9-42.7) | 36.2 (33.7-40.2) |
| DT | 39 (31-51) | 35.5 (30-51) | 28 (23-44) | 37.8 (31.4-44.7) | 35.7 (28-38.9) | 36.9 (30.3-42.0) |
| DBT | 40.5 (30-51) | 37 (26-47) | 31 (27-50) | 37.4 (31.8-45.3) | 36.6 (33.3-36.9) | 37.6 (34.6-39.5) |
| DBP | 40 (29-44) | 32 (23-51) | 30 (28-35) | 37.9 (35.3-42) | 34.6 (32.8-39.5) | 36.9 (33.3-45.1) |
| DK | 40 (37-46) | 35.5 (32-43) | 35 (30-38) | 39.6 (37.1-44.8) | 35.5 (29.3-36.7) | 36 (33.6-40.5) |
| DM | 41 (36-56) | 34.5 (32-44) | 34.5 (27-39) | 37.7 (28.8-44.7) | 34.9 (31.9-42) | 35.1 (30.9-40.4) |

Glu: glucose concentration (mg/dL), $pO_2$ and $pCO_2$ are measured in mmHg.
$pO_2$: partial pressure of oxygen in venous blood.
$pCO_2$: partial pressure of carbon dioxide in venous blood.
T0: baseline-dexmedetomidine administration.
T1: maximum sedation with dexmedetomidine-2$^{nd}$ drug administration.
T2: maximum sedation with the drug combination.
Group DD: administration of two repeated doses of dexmedetomidine; DC: dexmedetomidine-NS 0.9% combination (control group); DT: dexmedetomidine–tramadol combination; DBT: dexmedetomidine–butorphanol combination; DBP: dexmedetomidine–buprenorphine combination; DK: dexmedetomidine–ketamine combination; DM: dexmedetomidine–midazolam combination.

**Table S2.** Comparison of the median pH values between time points T0 and T2, in the seven groups of adult cats that received different drug combinations.

| GROUP | T0-T2 (p value) |
|---|---|
| DD | (0.497) |
| DC | Coef.: 0.048 (0.002) |
| DT | Coef.: 0.047 (0.001) |
| DBT | (0.093) |
| DBP | Coef.: 0.035 (0.018) |
| DK | Coef.: 0.06 (<0.001) |
| DM | Coef.: 0.037 (0.009) |

If the difference is statistically significant then, the co-efficiency (Coef.) value is also presented. A Coef.>0 indicates that the pH value was significantly higher at T2 in comparison to T0. All Confidence Intervals were set at 95%. The p values are presented in the parentheses.
T0: baseline, dexmedetomidine administration.
T1: maximum sedation with dexmedetomidine-2nd drug administration.
T2: maximum sedation with the drug combination and administration of atipamezole.
Group DD: administration of two repeated doses of dexmedetomidine; DC: dexmedetomidine-NS 0.9% combination (control group); DT: dexmedetomidine–tramadol combination; DBT: dexmedetomidine–butorphanol combination; DBP: dexmedetomidine–buprenorphine combination; DK: dexmedetomidine–ketamine combination; DM: dexmedetomidine–midazolam combination.

**Table S3.** Comparison of the median $pCO_2$ values between the time intervals, in the seven groups of adult cats that received different drug combinations.

| GROUP | T1-T0 (p value) | T1-T2 (p value) | T0-T2 (p value) |
|---|---|---|---|
| DD | (0.562) | (0.322) | (0.567) |
| DC | (0.539) | (0.897) | (0.496) |
| DT | (0.274) | (0.812) | (0.317) |
| DBT | (0.711) | Coef.: 0.9 (<0.001) | (0.871) |
| DBP | (0.435) | (0.263) | (0.921) |
| DK | (0.162) | (0.668) | (0.094) |
| DM | (0.857) | (0.683) | (0.798) |

$pCO_2$ is measured in mmHg. If the difference is statistically significant then, the co-efficiency (Coef.) value is also presented. The Coef.>0 indicates that the $pCO_2$ value was significantly higher at T2 in comparison to T1. All Confidence Intervals were set at 95%. The p values are presented in the parentheses.
T0: baseline, dexmedetomidine administration.
T1: maximum sedation with dexmedetomidine-2nd drug administration.
T2: maximum sedation with the drug combination and administration of atipamezole.
Group DD: administration of two repeated doses of dexmedetomidine; DC: dexmedetomidine-NS 0.9% combination (control group); DT: dexmedetomidine–tramadol combination; DBT: dexmedetomidine–butorphanol combination; DBP: dexmedetomidine–buprenorphine combination; DK: dexmedetomidine–ketamine combination; DM: dexmedetomidine–midazolam combination.

**Table S4.** Comparison of the median Glu values between T1 and T2 and between T0 and T2, in the seven groups of adult cats that received different drug combinations.

| **GROUP** | **T1-T2** (p value) | **T0-T2** (p value) |
|---|---|---|
| DD | Coef.: 36 (0.028) | Coef.: 77 (<0.001) |
| DC | Coef.: 33 (0.045) | (0.056) |
| DT | Coef.: 70 (0.009) | Coef.: 89 (0.002) |
| DBT | Coef.: 32 (0.032) | Coef.: 46 (0.002) |
| DBP | Coef.: 41 (0.003) | Coef.: 69 (<0.001) |
| DK | Coef.: 63 (<0.001) | Coef.: 71 (<0.001) |
| DM | Coef.: 49 (0.005) | Coef.: 56 (0.001) |

Glu concentration is measured in mg/dL. If the difference is statistically significant then, the co-efficiency (Coef.) value is also presented. The Coef.>0 indicates that the $pCO_2$ value was significantly higher at T2 than at T1 or the $pCO_2$ value was significantly higher at T2 than at T0. All Confidence Intervals were set at 95%. The p values are presented in the parentheses.
T0: baseline, dexmedetomidine administration.
T1: maximum sedation with dexmedetomidine-2$^{nd}$ drug administration.
T2: maximum sedation with the drug combination and administration of atipamezole.
Group DD: administration of two repeated doses of dexmedetomidine; DC: dexmedetomidine-NS 0.9% combination (control group); DT: dexmedetomidine–tramadol combination; DBT: dexmedetomidine–butorphanol combination; DBP: dexmedetomidine–buprenorphine combination; DK: dexmedetomidine–ketamine combination; DM: dexmedetomidine–midazolam combination.

**Table S5.** Comparison of the mean K+ concentrations at time point T1 between the seven groups of adult cats that received different drug combinations.

| GROUP | DD (p value) | DC (p value) | DT (p value) | DBT (p value) | DBP (p value) | DK (p value) |
|---|---|---|---|---|---|---|
| DC | Coef.: 0.51 (0.016) | | | | | |
| DT | (0.120) | (0.392) | | | | |
| DBT | (0.052) | (0.641) | (0.697) | | | |
| DBP | (0.062) | (0.586) | (0.756) | (0.938) | | |
| DK | (0.276) | (0.186) | (0.641) | (0.392) | (0.437) | |
| DM | Coef.: 0.45 (0.036) | (0.756) | (0.586) | (0.876) | (0.815) | (0.312) |

If the difference is statistically significant then, the co-efficiency (Coef.) value is also presented. A Coef. >0 indicates that the K+ concentration was significantly higher for the group in the row than that for the group in the column. A Coef. <0 indicates that the K+ concentration was significantly lower for the group in the row than for the group in the column. All Confidence Intervals were set at 95%. The p values are presented in the parentheses. K+ concentration is measured in mEq/L.

T2: maximum sedation with the drug combination and administration of atipamezole.
Group DD: administration of two repeated doses of dexmedetomidine; DC: dexmedetomidine-NS 0.9% combination (control group); DT: dexmedetomidine–tramadol combination; DBT: dexmedetomidine–butorphanol combination; DBP: dexmedetomidine–buprenorphine combination; DK: dexmedetomidine–ketamine combination; DM: dexmedetomidine–midazolam combination.

**Table S6.** Comparison of the median HCT values between the time intervals, in the seven groups of adult cats that received different drug combinations.

| GROUP | **T1-T0** (p value) | **T1-T2** (p value) | **T0-T2** (p value) |
|---|---|---|---|
| DD | 0.125 | 0.8125 | 0.0625 |
| DC | 0.0625 | 0.5625 | **0.0313** |
| DT | 0.0625 | 0.5313 | **0.0313** |
| DBT | **0.0313** | 0.500 | **0.0313** |
| DBP | 0.0938 | 0.0938 | **0.0313** |
| DK | **0.0313** | 0.625 | **0.0313** |
| DM | 0.0625 | 0.875 | 0.0625 |

All Confidence Intervals were set at 95%. The p values are presented in the parentheses.
T0: baseline, dexmedetomidine administration.
T1: maximum sedation with dexmedetomidine-2nd drug administration.
T2: maximum sedation with the drug combination and administration of atipamezole.
Group DD: administration of two repeated doses of dexmedetomidine; DC: dexmedetomidine-NS 0.9% combination (control group); DT: dexmedetomidine–tramadol combination; DBT: dexmedetomidine–butorphanol combination; DBP: dexmedetomidine–buprenorphine combination; DK: dexmedetomidine–ketamine combination; DM: dexmedetomidine–midazolam combination.

**Table S7.** Comparison of the median Hb concentrations between the time intervals, in the seven groups of adult cats that received different drug combinations.

| GROUP | **T1-T0** (p value) | **T1-T2** (p value) | **T0-T2** (p value) |
|---|---|---|---|
| DD | 0.125 | 0.3125 | 0.0625 |
| DC | 0.0625 | 0.6250 | **0.0313** |
| DT | 0.0625 | 0.4688 | **0.0313** |
| DBT | **0.0313** | 0.0625 | **0.0313** |
| DBP | **0.0313** | 0.1875 | **0.0313** |
| DK | **0.0313** | 0.625 | **0.0313** |
| DM | 0.0625 | 0.9375 | 0.0625 |

All Confidence Intervals were set at 95%. The p values are presented in the parentheses.
T0: baseline, dexmedetomidine administration.
T1: maximum sedation with dexmedetomidine-2nd drug administration.
T2: maximum sedation with the drug combination and administration of atipamezole.
Group DD: administration of two repeated doses of dexmedetomidine; DC: dexmedetomidine-NS 0.9% combination (control group); DT: dexmedetomidine–tramadol combination; DBT: dexmedetomidine–butorphanol combination; DBP: dexmedetomidine–buprenorphine combination; DK: dexmedetomidine–ketamine combination; DM: dexmedetomidine–midazolam combination.

**Table S8.** Median (range) HR and RR at T0, T1, T2, and T3 for the seven groups of six adult cats that received different drug combinations.

| | **HR** | | | |
|---|---|---|---|---|
| **Groups** | **T0** | **T1** | **T2** | **T3** |
| DD | 150.7 (120-184) | 97 (90-108) | 89 (81-99) | 137 (108-188) |
| DC | 180 (150-200) | 126.3 (105-164) | 124.5 (99-140) | 150 (123-180) |
| DT | 181.3 (160-200) | 108.7 (88-120) | 105.7 (99-118) | 154 (132-180) |
| DBT | 183.5 (150-220) | 114-7 (102-132) | 96 (81-105) | 149.5 (139-181) |
| DBP | 181.7 (148-200) | 122.3 (92-154) | 114.2 (100-147) | 155 (128-180) |
| DK | 165.8 (120-200) | 126.8 (99-160) | 130 (112-176) | 163.5 (150-190) |
| DM | 178.3 (150-220) | 115 (90-128) | 114 (100-136) | 150 (128-152) |
| | **RR** | | | |
| **Groups** | **T0** | **T1** | **T2** | **T3** |
| DD | 51.5 (30-96) | 38 (24-54) | 41 (33-54) | 42 (33-60) |
| DC | 54 (42-75) | 40 (30-45) | 45.5 (33-54) | 42 (36-60) |
| DT | 51 (45-63) | 41 (27-51) | 41.5 (39-48) | 57 (45-69) |
| DBT | 62.2 (39-96) | 46 (33-60) | 34.5 (30-45) | 57 (42-60) |
| DBP | 49.5 (21-63) | 43.5 (30-54) | 40 (33-51) | 42 (33-57) |
| DK | 47 (36-69) | 40.5 (33-48) | 40.5 (33-51) | 39 (33-54) |
| DM | 57.7 (51-64) | 52.5 (45-63) | 42.5 (36-51) | 43.5 (33-60) |

HR: heart rate (beats per min - bpm).
RR: respiratory rate (breaths per min - bpm).
T0: baseline, dexmedetomidine administration.
T1: maximum sedation with dexmedetomidine-2nd drug administration.
T2: maximum sedation with the drug combination and administration of atipamezole.
T3: full recovery.
Group DD: administration of two repeated doses of dexmedetomidine; DC: dexmedetomidine-NS 0.9% combination (control group); DT: dexmedetomidine–tramadol combination; DBT: dexmedetomidine–butorphanol combination; DBP: dexmedetomidine–buprenorphine combination; DK: dexmedetomidine–ketamine combination; DM: dexmedetomidine–midazolam combination.

**Table S9.** Comparison of the median HR values at time point T1 between the seven groups of adult cats that received different drug combinations.

| **GROUP** | **DD** (p value) | **DC** (p value) | **DT** (p value) | **DBT** (p value) | **DBP** (p value) | **DK** (p value) |
|---|---|---|---|---|---|---|
| DC | Coef.: -30 (0.005) | | | | | |
| DT | (0.423) | (0.057) | | | | |
| DBT | Coef.: -18 (0.02) | (0.337) | (0.405) | | | |
| DBP | Coef.: -24 (<0.001) | (0.446) | (0.121) | (0.584) | | |
| DK | Coef.: -27 (0.011) | (0.733) | (0.101) | (0.309) | (0.784) | |
| DM | (0.114) | (0.06) | (0.309) | (1.00) | (0.529) | (0.153) |

If the difference is statistically significant then, the co-efficiency (Coef.) value is also presented. A Coef.>0 indicates that the HR value was significantly higher for the group in the row than that for the group in the column. A Coef.<0 indicates that the HR value was significantly lower for the group in the row than for the group in the column. All Confidence Intervals were set at 95%. The p values are presented in the parentheses.

T1: maximum sedation with dexmedetomidine-2nd drug administration.
Group DD: administration of two repeated doses of dexmedetomidine; DC: dexmedetomidine-NS 0.9% combination (control group); DT: dexmedetomidine–tramadol combination; DBT: dexmedetomidine–butorphanol combination; DBP: dexmedetomidine–buprenorphine combination; DK: dexmedetomidine–ketamine combination; DM: dexmedetomidine–midazolam combination.

**Table S10.** Comparison of the median RR values at time point T1 between the seven groups of adult cats that received different drug combinations.

| GROUP | DD (p value) | DC (p value) | DT (p value) | DBT (p value) | DBP (p value) | DK (p value) |
|---|---|---|---|---|---|---|
| DC | (0.500) | | | | | |
| DT | (0.592) | (1.00) | | | | |
| DBT | (0.164) | (0.579) | (0.660) | | | |
| DBP | (0.578) | (1.00) | (1.00) | (0.702) | | |
| DK | (0.681) | (0.614) | (0.614) | (0.337) | (0.724) | |
| DM | (0.094) | (0.086) | Coef.: 12 (0.018) | (0.649) | (0.106) | Coef.: 12 (0.009) |

RR is measured in breaths per minute.
If the difference is statistically significant then, the co-efficiency (Coef.) value is also presented. A Coef.>0 indicates that the RR value was significantly higher for the group in the row than that for the group in the column. A Coef.<0 indicates that the RR value was significantly lower for the group in the row than for the group in the column. All Confidence Intervals were set at 95%. The p values are presented in the parentheses.
T1: maximum sedation with dexmedetomidine-2nd drug administration.
Group DD: administration of two repeated doses of dexmedetomidine; DC: dexmedetomidine-NS 0.9% combination (control group); DT: dexmedetomidine–tramadol combination; DBT: dexmedetomidine–butorphanol combination; DBP: dexmedetomidine–buprenorphine combination; DK: dexmedetomidine–ketamine combination; DM: dexmedetomidine–midazolam combination.

**Table S11.** Comparison of the median HR values between time points T1 and T0 and between T0 and T2, in the seven groups of adult cats that received different drug combinations.

| GROUP | T1-T2 (p value) | T0-T2 (p value) |
|---|---|---|
| DD | (0.363) | Coef.: 60 (0.007) |
| DC | (1.00) | Coef.: 54 (0.002) |
| DT | (1.00) | Coef.: 75 (<0.001) |
| DBT | (0.275) | Coef.: 90 (0.002) |
| DBP | (0.503) | Coef.: 79 (<0.001) |
| DK | (0.854) | (0.083) |
| DM | (0.682) | Coef.: 92 (0.001) |

HR is measured in beats per minute.
If the difference is statistically significant then, the co-efficiency (Coef.) value is also presented. A Coef.>0 indicates that the HR value was significantly higher at T0 than at T2. All Confidence Intervals were set at 95%. The p values are presented in the parentheses.

T0: baseline, dexmedetomidine administration.
T1: maximum sedation with dexmedetomidine-2$^{nd}$ drug administration.
T2: maximum sedation with the drug combination and administration of atipamezole.
Group DD: administration of two repeated doses of dexmedetomidine; DC: dexmedetomidine-NS 0.9% combination (control group); DT: dexmedetomidine–tramadol combination; DBT: dexmedetomidine–butorphanol combination; DBP: dexmedetomidine–buprenorphine combination; DK: dexmedetomidine–ketamine combination; DM: dexmedetomidine–midazolam combination.
